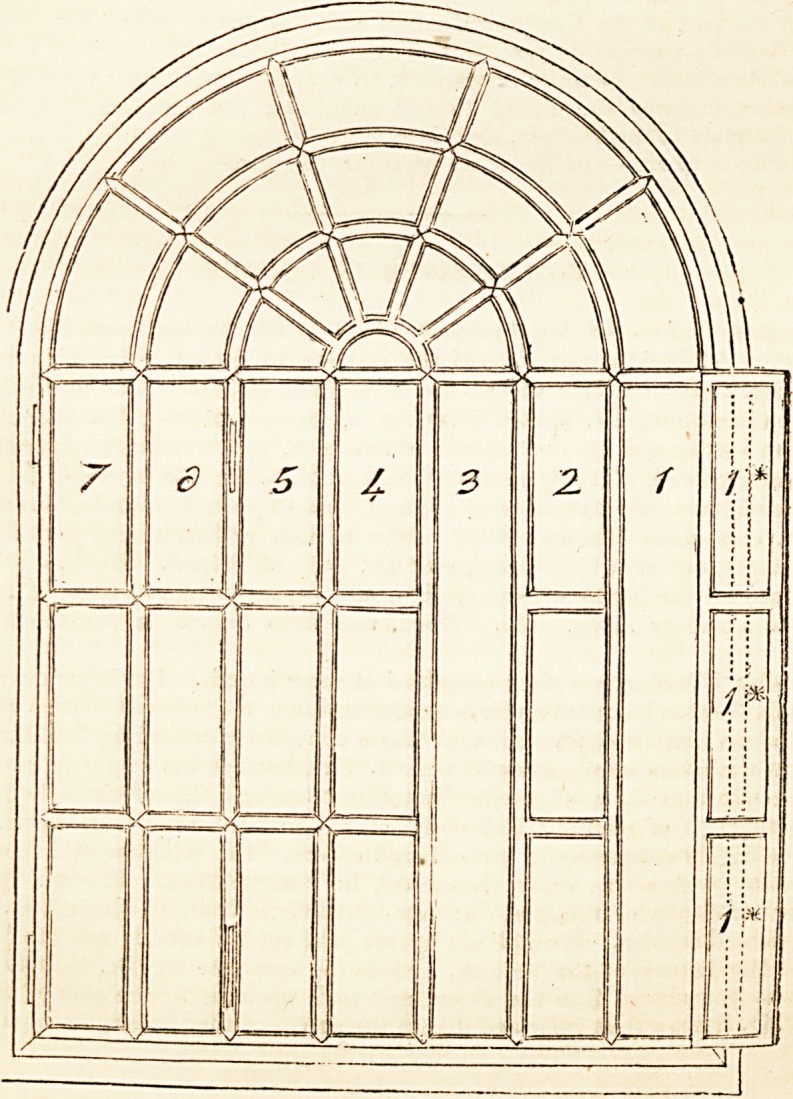# Description of a New Window for the Use of Asylums

**Published:** 1851-01-01

**Authors:** W. Wood

**Affiliations:** LICENTIATE OF THE ROYAL COLLEGE OF PHYSICIANS; RESIDENT MEDICAL OFFICER OF BETHLEM HOSPITAL


### DESCRIPTION OF A NEW WINDOW FOR THE USE OF ASYLUMS.

BY W. WOOD, M.D. LICENTIATE OF THE ROYAL COLLEGE OF PHYSICIANS; RESIDENT MEDICAL OFFICER OF BETHLEM HOSPITAL.

WHENEVER the stern necessity arises for breaking up a family circle, and consigning to an asylum some one of its members, the first thought which the anxiety of relatives properly and naturally suggests is, how the distress occasioned by the separation of those who have perhaps never yet been parted may be mitigated, if not entirely saved—how the gloomy presentiments of coming evils, and the distracting doubts of groundless suspicions, may be most speedily removed, while the wild excitement of mania may be calmed with the least *appearance* of opposition or control. The very aspect of the building to which a patient is often so unwillingly taken, may make an impression on his mind most difficult to efface, and most prejudicial to his recovery. Important as they are, it is not enough to provide for the mere animal wants and personal comforts, nor will it suffice to insure the kind assiduity of attendants, and all the resources which various well-ordered amusements afford, if the arrangements of the building itself suggest the thought of imprisonment, and remind the unhappy sufferer, as he wistfully gazes through his heavily-barred window, that he is not only forcibly separated from his family, but at the same time shut out from the world. I need not dwell on the impediments to recovery which all unnecessary precautions are so well calculated to produce; they not only tend to perpetuate gloomy thoughts, to create discontent, and to rouse opposition, but are constantly referred to as arguments in proof of some imaginary wickedness already committed, or some still more severe punishment yet to be endured. Universal anxiety is manifested on the part of all those concerned in the erection and management of asylums, to deprive them as much as possible of all prison-like appearance, and to imprint on them, as it were, the aspect of cheerfulness. The importance of appearances has not been lost sight of; and though the windows have received a fair share of consideration, and many great improvements have been effected in their construction, the great desideratum remains yet to be supplied—viz., a window which shall allow the greatest extent of free and uninterrupted circulation of air without the appearance of bars or other contrivances, which are obviously intended only for security. The windows are the objects which most frequently remind the captive of his condition, and tell him, in no very gentle terms, that he may not be trusted with the same liberty as his fellow-men. But, independently of the depressing effect of such means of security as are frequently out of all proportion to what is really required, there is something extremely irritating to sensitive minds in being doomed continually to look through bars, which are obviously *only* placed there for the safe keeping of the inmates. The governors of Bethlem Hospital having turned their attention seriously to the improvement of the windows, with a view to remove all unnecessary appearance of security, and so contribute to the cheerfulness of the galleries, and the lighter aspect from without, caused model windows to be constructed and fixed, for the purpose of comparing the relative advantages of different designs. The first consisted of an iron window, cast in three pieces, the top, which is necessarily arched, to suit the original plan, being fixed—the two other portions being so constructed as to swing horizontally on a central axis, after the plan of luffer boards, only, of course, not overlapping each other, but, when shut, fitting so closely as not to show any increased thickness of the bar, which divides when the window opens. As regards appearance when closed, this plan is consistent with everything that can be desired, but there are some objections to it: in the first place, the contrivance for opening it, which consists of a moving rack, worked by a key, is somewhat complicated, and requires the expenditure of some force and time; and this, multiplied over a number of windows, would amount to something rather considerable. A greater objection still is, that it does not open sufficiently wide for the purpose of ventilation, and the patients could not very well be left to open it themselves. Two other model windows are simply modifications of a principle already adopted—viz., a wooden sash, to open as a French window, with an iron guard, corresponding to the divisions of the wooden sash, which is made to shut so close on to the iron as to appear like one bar. Here, again, there is nothing very objectionable in the appearance while shut, excepting that the total depth, resulting from the combined thickness of the wooden and iron bars, looks heavy; but when open, the patient has to look through bars which are obviously only for the purpose of security, and therefore necessarily suggestive of the circumstances which require such contrivances.

Whatever may be the artificial means of ventilation employed in an asylum, it is desirable, at any rate, to be able to open the windows as much as possible, consistent with safety—in other words, leaving only such spaces as will not enable a patient to escape through; and it is also desirable that the opening and shutting should be as simple as possible. Next in importance to the necessary security comes the appearance, both within and without; and the importance of this consideration can, I think, scarcely be over-estimated.

It occurred to me that a window might be contrived, sufficiently secure, without any appearance of bars, which would allow of a considerable space being entirely open, as much, in fact, or even more, than could be obtained in the case of an ordinary house-window, where one sash slides or is pulled over the other, and the greatest amount of open space is obtained when the two sashes occupy the same level, and just half of the whole opening occupied by the window is without any interruption to the free circulation of air. The first question, then, was to decide what was the greatest width of pane that could be allowed consistently with safety. I found, on measuring a large number of heads, that the broadest part of any as young as twelve years of age, was something more than 5½ inches, and I therefore determined to adopt that measurement for my design. The length of pane is quite immaterial, and is merely a question of some trifling additional expense in glazing; but, as far as appearances go, the longer it is the better, of course within reasonable limits, and having regard to the size of the whole window, its necessary divisions, and the general style of the building. The principal advantage of long panes is, that they diminish the number of divisions, and consequently show the least possible quantity of iron. The next important part of the design is the manner of opening, which I conceive should be as simple as possible, so that the patients themselves may at any time, without difficulty, open a window when they wish it, and having opened it, find nothing suggestive of the control to which they are subjected: this semblance of liberty, even in such an apparently trifling matter, is well worth providing for. When the intention of accomplishing security is not apparent, there is less danger of arousing a spirit of resistance, and patients are more likely to submit quietly to arrangements which appear, after all, to differ so little from those in their own homes. But I have said, that having opened a window, the patient should see nothing suggestive of the control to which he is subjected. This is accomplished by opening the whole length of the window to the extent of one pane in width in one piece, as shown in the annexed sketch. The most external portions are folded back, quite out of the way, against the wall of either side; the other portions of the window which open are folded back upon the adjoining compartments, and are made to fit so closely as to appear one with the frame on to which they fold; the most important, and I believe original, part of the design being, that no bar appears in any of these open spaces, though four-sevenths of the whole space, if we except the top—which may or may not be fixed—is entirely open; if the window were square at the top, four-sevenths of the whole space might be entirely open. The design supposes the top of the window fixed; but it might be made to open entirely in one piece by means of a hinge, or in compartments; and this might be necessary, if it were adopted for windows in any story above the ground-floor, on account of the inconvenience which would arise in cleaning or repairing; the former might be accomplished altogether from within, perhaps without any other than the lower part of the window opening, but at any rate by the top pane—corresponding as it were to the key-stone of the arch, being made to fall on a hinge or hinges. As regards repairs, the lower part of the windows, when they are most likely to be required, opens sufficiently to enable them to be done entirely from within, and if the top of the window is square, the same principle of opening would extend to the top and remove all difficulty on this score, but if arched, an accident is so rare in this situation as really to render the difficulty unimportant, and a ladder would always reach from the outside, if the opening of the top frame were not sufficient. It is perhaps sufficient to say of the fastenings, that if any are really necessary, they may be of the simplest possible construction, perhaps a button, just to prevent the wind blowing the window open. Under ordinary circumstances, it is presumed that if the window be properly made, its own weight would keep it shut.

The favourable opinion that has been expressed of the model window, and the declared intention of our architect, who has seen it, to adopt it in a county asylum which he is now building, has induced me to draw up this description of it for the *Psychological Journal,* under the impression that it may be thought worthy of the consideration of those who are interested in the improvement and construction of asylums.

### Description of the Plate.

The accompanying sketch represents the window as seen from within, one side being fully opened, the other entirely closed. The compartments marked with odd numbers, viz. 1, 3, 5, and 7, are those which open; those marked with even numbers, viz. 2, 4, and 6, are fixed, and do not open. The spaces 1 and 3 are represented open, 1 being folded back against the wall, 3 being folded back upon 2, which, though now really double, is intended to have the same appearance as when single. The compartments which may yet be opened are 5 and 7, and these would fold back, the former upon 6, the latter against the wall. It will of course be understood, that each compartment which opens is in one piece, as seen at 1*, and the whole security of the window depending upon the bars, which are fixed, the moveable frame need not be heavy—indeed only strong enough to carry the glass.

## Figures and Tables

**Figure f1:**